# Evolving Adult ADHD Care: Preparatory Evaluation of a Prototype Digital Service Model Innovation for ADHD Care

**DOI:** 10.3390/ijerph21050582

**Published:** 2024-05-01

**Authors:** Bronwin Patrickson, Lida Shams, John Fouyaxis, Jörg Strobel, Klaus Oliver Schubert, Mike Musker, Niranjan Bidargaddi

**Affiliations:** 1 Digital Health Research Lab, College of Medicine and Public Health, Flinders University, Adelaide 5042, Australia; lida.shams@flinders.edu.au (L.S.); john.fouyaxis@flinders.edu.au (J.F.); jorg.strobel@sa.gov.au (J.S.); 2 Division of Mental Health, Barossa Hills Fleurieu Local Health Network, 29 North St, Angaston 5353, Australia; 3 Division of Mental Health, Northern Adelaide Local Health Network, 7-9 Park Terrace, Salisbury 5108, Australia; oliver.schubert@sa.gov.au; 4 Discipline of Psychiatry, Adelaide Medical School, The University of Adelaide, North Terrace, Adelaide 5005, Australia; 5 The Headspace Adelaide Early Psychosis, Sonder, 173 Wakefield St, Adelaide 5000, Australia; 6 Clinical Health Sciences, Mental Health and Suicide Prevention Research and Education Group, University of South Australia, City East, Centenary Building, North Terrace, Adelaide 5000, Australia; mike.musker@unisa.edu.au

**Keywords:** adult ADHD, digital service innovation, mHealth, self-monitoring, self-management, clinical decision support tools, clinical support tools

## Abstract

Background: Given the prevalence of ADHD and the gaps in ADHD care in Australia, this study investigates the critical barriers and driving forces for innovation. It does so by conducting a preparatory evaluation of an ADHD prototype digital service innovation designed to help streamline ADHD care and empower individual self-management. Methods: Semi-structured interviews with ADHD care consumers/participants and practitioners explored their experiences and provided feedback on a mobile self-monitoring app and related service innovations. Interview transcripts were double coded to explore thematic barriers and the enablers for better ADHD care. Results: Fifteen interviews (9 consumers, 6 practitioners) revealed barriers to better ADHD care for consumers (ignorance and prejudice, trust, impatience) and for practitioners (complexity, sustainability). Enablers for consumers included validation/empowerment, privacy, and security frameworks, tailoring, and access. Practitioners highlighted the value of transparency, privacy and security frameworks, streamlined content, connected care between services, and the tailoring of broader metrics. Conclusions: A consumer-centred approach to digital health service innovation, featuring streamlined, private, and secure solutions with enhanced mobile tools proves instrumental in bridging gaps in ADHD care in Australia. These innovations should help to address the gaps in ADHD care in Australia. These innovations should encompass integrated care, targeted treatment outcome data, and additional lifestyle support, whilst recognising the tensions between customised functionalities and streamlined displays.

## 1. Introduction

The approximate global prevalence of persistent adult Attention Deficit Hyperactivity Disorder (ADHD) is 2.58% (139.84 million), and that of symptomatic adult ADHD is 6.76% (366.33 million) [1]. The condition is marked by pronounced impulsivity, hyperactivity, and/or inattention which interferes with functioning according to the *Diagnostic and statistical manual of mental disorders: DSM-5-TR* [2], and can have profound impacts in multiple domains across a lifespan, including social and emotional well-being, relationships, academic and occupational outcomes, physical and mental health, quality of life, and even life expectancy [3,4]. Although the awareness of adult ADHD has increased over time [5,6,7,8,9], numerous barriers to care nevertheless persist. Many adults with ADHD go undiagnosed [10,11,12,13]. Of those who do receive a diagnosis, only one quarter receive treatment. Against the backdrop of a historic debate in regards to the prevalence and ideal care for this condition [14], the diagnosis and treatment of adult ADHD involves numerous challenges both in Australia [15,16] and worldwide [13,17,18].

In Australia, since ADHD is commonly treated with psychostimulants, such as methylphenidate, or dexamphetamine, the diagnosis and treatment of the condition is strictly regulated and shared between General Practitioners (GPs) and psychiatrists. According to controlled medicine use regulations, GPs must refer potential ADHD care consumers for formal assessment by a qualified psychiatrist, and any administered pharmacological treatments are closely monitored by government oversight bodies. However, there are various health service factors that worsen accessibility to ADHD diagnosis and treatment. These include the limitations of clinical resources within the healthcare system, lengthy diagnostic procedures, and a noticeable lack of specialised expertise tailored to meet distinct patient needs [19,20,21,22]. Additionally, there is a concern that the long-term side effects of pharmacological treatments, such as the development of tics and sleep disorders over time [23] or stimulant induced psychosis [24], are not being effectively monitored. Moreover, ADHD, as a multifaceted condition, often responds best to a fusion of multimodal treatment approaches [25], such as pharmacotherapy and psychotherapy [26], yet such treatment strategies are not always harmoniously integrated.

Given the profound personal, familial, and societal costs [27] of untreated ADHD, it is critical to ensure the early detection and effective treatment of this disorder. To address these challenges and to improve treatment outcomes for individuals with ADHD; networked, affordable, and accessible digital health interventions offer a promising solution. Mobile health (mHealth) programs are gaining prominence in this regard [28]. The rise in mobile phone usage has opened opportunities for mHealth apps, which have the potential to overcome certain constraints associated with traditional therapy, such as costly treatments and limited access to treating psychiatrists [28,29,30,31,32,33]. As a result of their digital capacities, mHealth apps can offer customisable and targeted services like automated medication reminders that are accessible anywhere and at any time, or streamlined health indicator monitors and patient-specific assessments and recommendations that can then be presented to clinicians as a decision support aid. Such systems have been shown to improve prescribing practices, reduce serious medication errors, enhance the delivery of preventative care services, and improve adherence to recommended care. In a systematic review of 70 clinical studies, decision support systems significantly improved clinical practice in 68% of trials [34]. Networked decision support systems also enable information exchange within the clinical team, which assists in managing demand for health services and lowering direct medical costs for consumers.

Despite the potential benefits of mHealth apps in enabling more accessible, flexible, and responsive ADHD care, there remains a research-to-practice gap, illustrated by low uptake rates of mHealth mental health apps. This is often due to a lack of participatory stakeholder involvement in the design of mHealth apps, which as a result, “do not fit into clinicians’ workflows and are not aligned with how patients want to use personal technologies” [33:88]. Such oversights are reflected in two previous reviews of available apps for young people with ADHD. A 2020 systematic review of 109 mobile apps designed for ADHD listed in app stores found that none contained information on an evidence basis [35], highlighting the need for expert involvement and validating content to ensure a quality service [36]. Additionally, a study of the top 10 listed mobile apps targeting young people with ADHD found that they could be more engaging (inviting, relatable, motivational and potentially rewards based), and easier to use, and were unlikely to meet the complex needs of this consumer cohort [37].

Scant research exists on the needs and perspectives of adult Australian ADHD consumers and health professionals regarding the potential contribution of mHealth interventions for clinical adult ADHD care. Globally, a small number of feasibility studies on mobile lifestyle aids and psychoeducation supports have concluded that there is a significant demand for such apps when they are designed optimally and in collaboration with participants [38,39]. Additionally, a handful of random control trials have concluded that on-demand psychoeducation is more effective when delivered by a smartphone rather than traditional brochures [40]. Furthermore, smartphones have been identified as offering effective alternative delivery pathways for psychoeducational therapy, including self-guided ADHD psychoeducation based on a chatbot or conventional app [40], or else in combination with physiological health data monitoring (via a wrist-worn sensor) and telehealth coaching sessions [41]. Studies of smartphone-assisted participant self-monitoring of ADHD pharmacological outcomes are even rarer. One pilot study of SMS-based monitors has concluded that participants find them easy to engage with and prefer them to paper and pen diary monitors [42].

Many mHealth design and development frameworks still focus on the underlying software or apps as a stand-alone tool [43], rather than frame those tools as an integral element of a broader service innovation project [44]. However, we argue that the successful implementation and adoption of mHealth tools for ADHD care depends equally upon their integration with relevant social, cultural, organisational and legal contexts [45,46,47,48]. Therefore, this study attempts to provide insights from both consumers and health professionals (i.e., ADHD mHealth service stakeholders) in the context of an integrated reconfiguration of ADHD health care services. To do so, it applies service dominant (S-D) logic [49], which emphasises that mHealth apps are not only innovative goods or tools produced by service providers, but rather interactive enablers of potentially transformative service model innovations [50,51]. This approach frames health care service provision as a process of exchange and value co-creation that uses specialised resources for the benefit of stakeholders [49].

### 1.1. The Prototype Service Innovation

To help improve ADHD care, this preparatory study [52] explores stakeholder needs, ADHD health service gaps, and the potential barriers and enablers of a prototype digital health ADHD care service model innovation. This prototype service innovation is designed with the view to transform ADHD care post-diagnosis. It includes an mHealth clinical decision support tool and clinician overview dashboard spearheaded by a mobile app which offers self-monitored assessments and psychoeducation tools to enable ADHD care consumers to self-manage their wellbeing and monitor treatment outcomes more effectively through regular targeted self-check-ins. The prototype is a multi-site ADHD service innovation developed by goAct Pty Ltd. (Adelaide, South Australia); a digital health technology provider led by a psychiatrist who treats ADHD care consumers. goAct Pty Ltd. has previously developed and hosted a suite of consumer mobile self-monitoring applications and partner clinician dashboards for a variety of health conditions. The service innovation is ultimately intended to be deployed in multiple and varying ADHD care provision sites. Beyond simply testing a mobile app and partner clinician dashboard, this preparatory study considers service innovation needs more generally, such as the need for potential support services, like ADHD self-management coaching, and optimal ways to provide clinical oversight of self-monitored wellbeing data in the ADHD health care service model.

The app itself monitors the user’s condition in real-time, evaluating their mental and emotional well-being across eight specific domains. Two of these are directly related to ADHD—Impulsivity/Hyperactivity and Focus—while the others encompass Anxiety, Stress, Mood, Sleep and Energy, Confidence, and Relationships. Utilising Ecological Momentary Assessments, [53,54] the app provides short targeted questions at random moments of time, providing real-time treatment recommendations, and promoting interactions between care consumers and clinicians. This enquiry method embeds monitoring in daily life and can effectively gauge the user’s state of mind and emotional health with accuracy rates, which are comparable with more extensive questionnaires [55]. This method, rooted in the foundational work of Shiffman and Stone [53,54], employs frequent sampling to quickly and accurately assess an individuals’ behavioural and cognitive processes within their natural day-to-day environments, offering a comprehensive view of their ongoing mental and emotional state.

### 1.2. Research Aims and Objectives

This study seeks to identify the ways the components of the ADHD service innovation can potentially intertwine with and transform existing ADHD care models. It seeks to accomplish this by identifying the potential barriers and enablers for implementing the mobile self-monitoring app ADHD and related service model innovations. To assess further needs for adaptation and development, this research probes the following:

(1) Identifying barriers and enablers for ADHD health service innovation;
(2) Understanding how this digital intervention can impact the clinical experience, and;
(3) Assessing the acceptability of individual features of the prototype ADHD service innovation in enhancing ADHD management.

In summary, this study emphasises the importance of improving clinical decision support for ADHD and empowering consumers in self-management, exploring the use of mHealth tools and related mHealth service innovations designed to help address these challenges.

## 2. Materials and Methods

This study employed a qualitative research approach to gain insights into human experiences and social contexts. Qualitative research is essential for uncovering the deeper meanings and perspectives that individuals associate with their lives [56]. For digital health service innovations to succeed, stakeholder guidance is generally crucial. Involving stakeholders authentically in co-design efforts can help to produce more acceptable and engaging interventions, offering increased utility for both recipients and providers [57]. Health care professionals’ perspectives are vital in improving existing tasks and maintaining evidence-based practices, but it is equally important to seek perspectives from individuals with ADHD—the potential care consumers who will be participating in the service innovation.

For this study, data were collected through semi-structured interviews, which allowed for a two-way communication environment and the exploration of participants’ beliefs and opinions. The study used a set of 16 questions for care consumers and 5 questions for practitioners to understand their experiences with ADHD and their potential engagement with various aspects of the ADHD Digital Service Innovation.

The questions were identified by the research team, in collaboration with research project industry partner goAct Pty Ltd.. In addition, these questions were mapped against relevant aspects of the updated consolidated framework for implementation research (CFIR) [58], which is a comprehensive implementation framework that can be used to help guide research into the potential barriers and enablers (or facilitators) of innovation. The updated CFIR is commonly utilized to assess numerous factors relevant to innovation implementation [59] and has been adapted for this research. Whereas the updated CFIR provides a comprehensive list of implementation considerations across numerous domains, our focus in this study was upon three of those areas:

(1) The characteristics of the digital ADHD care service innovation, including factors such as evidence strength and quality, and the usability and customisability of the app.
(2) The outer setting, which in this study encompasses ADHD treatment needs and resources.
(3) The characteristics of the potential participants of this innovation, such as their knowledge and attitudes.

**Figure 1:**
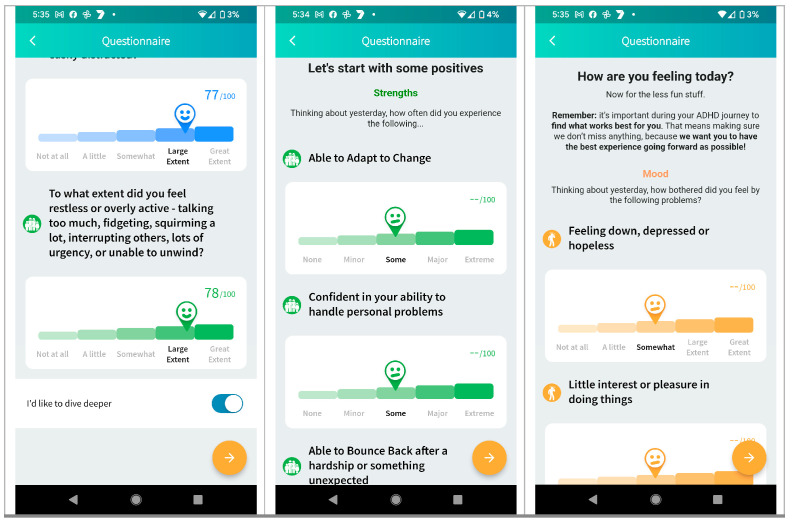
Example prototype mobile self-monitoring questionnaires shared with research participants during research interviews. Reproduced with permission from goAct Pty. Ltd.

**Figure 2:**
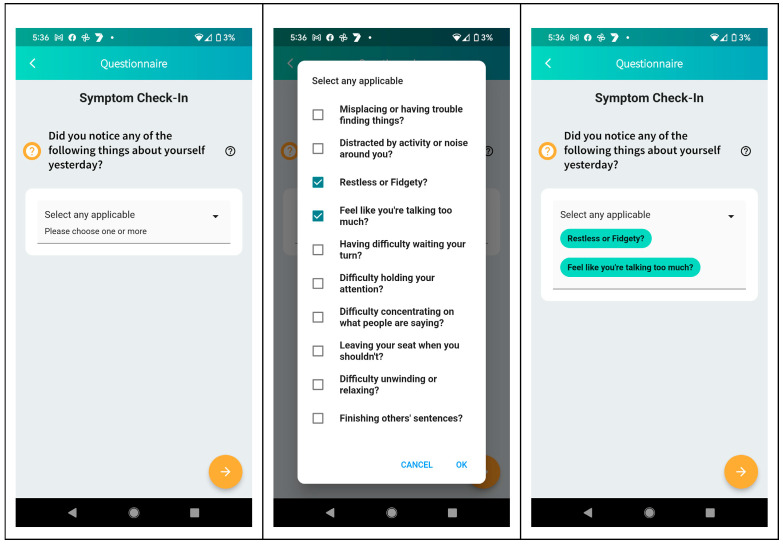
Example prototype mobile self-monitoring symptom checker questionnaires shared with research participants during research interviews. Reproduced with permission from goAct Pty. Ltd.

To review the characteristics of the digital ADHD care innovation, ADHD care consumer research participants and select ADHD practitioners were shown prototype display screens (see Figure 1 and Figure 2 Below), contextualized by introductory explanations of the nature and intent of that functionality:

Interviews, lasting 45 min on average, were conducted virtually and were recorded for accuracy. Two researchers used Nvivo 12 (Lumivero, Denver, CO, USA) to double code the transcripts and identify themes and patterns. Inductive analysis, as outlined by Braun and Clarke (2006), was employed to code and interpret the data without preconceived notions [60,61].

This method of content analysis was interpretive in nature. It aimed to describe or illuminate a phenomenon by examining both manifest content (the obvious) and latent content (underlying meaning) within a text. Here are the key steps involved:

1. Multiple Readings: Working independently to start, researchers read the text several times to become familiar with it and reflect on its content.
2. Identify Meaning Units: Within the text, segments were identified that described a phenomenon. These were the meaning units.
3. Assign codes: The essential content from the meaning units was highlighted and assigned relevant codes, illustrating the themes that they represented.
4. Theme Comparison: Researchers compared codes based on similarities and differences and organized them into themes. These themes covered various aspects, including barriers or enablers of the service innovation, interaction concerns, and visual design issues (such as clarity and ease of use).

The analysis, grounded in the data, aimed to identify the barriers and enablers of the ADHD service innovation, with differences resolved in discussions. The findings, discussed in the next section, reveal insights from participant responses and experiences. To help distinguish between discussions relating to contextual issues (the lived experience of ADHD diagnosis and care) and prototype design considerations, findings were divided between the identification of contextual barriers to better ADHD care, and potential enablers of the ADHD service innovation prototype itself. These agreed themes informed a range of prototype ADHD service innovation design and development recommendations.

### Sampling

This study defines the target consumer population as adults diagnosed with ADHD in Australia, referred to in this article as ADHD care consumers and/or service innovation participants. goAct Pty Ltd. facilitated the participant recruitment process by disseminating an email introduction to the study. Participants (including ADHD healthcare practitioners) were recruited through convenience sampling from goAct Pty Ltd.’s clinical networks, with recruitment taking place from 9 August to 9 October 2023. Consumer eligibility criteria included being 18 or older, having an ADHD diagnosis, experiencing daily functioning impairment, having access to a computer or smartphone with internet, and proficiency in English. Health professional eligibility criteria included professional experience with ADHD treatment and care, plus having access to a computer or smartphone with internet, and proficiency in English.

The study used a mixed purposive and convenience sampling approach, selecting individuals already connected to the technology partner’s established clinical networks with knowledge and experience relevant to the research questions. This ensured that participants could provide valuable insights based on their firsthand experience and substantial knowledge, aligned with the study’s objectives.

## 3. Results

In total, nine interviews with ADHD care consumers and six interviews with practitioners were conducted and are summarised in Table 1.

### 3.1. Consumer Discussion Themes

#### 3.1.1. Contextual Barriers to Better ADHD Care Identified by Consumers

A description of the main contextual barriers to better inform ADHD care identified by ADHD care consumers follows with example quotes in Table 2 below, including concerns about ignorance and prejudice, trust issues, and the potential for impatience associated with the ADHD condition.

- *Ignorance and Prejudice*

All care consumers in this study reported experiences of confusion and psychological distress because of long-standing ignorance about their ADHD condition. At least two participants had experienced substance use disorders, whilst others suffered from anxiety, and depression, all of which are common co-morbidities linked to ADHD [62]. The pain and shame of this experience commonly impacted participants’ self-esteem and limited their ability to seek help. Female participants often lacked knowledge of their condition during their formative years due to gender stereotypes, suggesting that ADHD was more prevalent in boys, which affected awareness. In total, 45% of consumer participants reported challenges in finding accurate information online and GP reactions to their ADHD concerns varied. Some GPs were supportive, whilst at least three consumer participants reported that their GP initially rejected the prospect of an ADHD diagnosis.

- *Trust*

Seven out of nine care consumer participants highlighted the sensitive nature of their health information, raising privacy and security concerns. One participant worried that the constant questions might cause them anxiety, and wanted reassurances about how the data would be used.

- *Impatience*

Seven out of nine participants expressed a sense of being overwhelmed, linked to the challenge of self-managing their ADHD and would welcome additional support in that area. Technical proficiency, as much as patience varied between participants, however. Whilst some already leant heavily upon mobile organisation assistance tools to help manage their ADHD, at least one participant proactively avoided technology and requested paper format alternatives. Nevertheless, even the use of lo-fi paper tools was likely to be plagued by the ‘focus and persistence’ challenges associated with the ADHD condition.

#### 3.1.2. Potential Enablers of the ADHD Service Innovation Identified by ADHD Care Consumers

As the example quotes in Table 3 below reflect, consumer feedback about the prototype ADHD service innovation implicates the potential enablers of better ADHD care for this participant group, which refers to the prevailing conditions and contextual factors likely to support the mHealth service innovation.

- *Validation/Empowerment*

As a result of their past experiences of ignorance and prejudice in regard to their ADHD condition, the importance of inclusive language, diagnostic validation, and clear clinical treatment pathways was repeatedly emphasised by participants.

Eight out of nine interviewees affirmed that a mobile self-monitoring/ADHD guidance tool had the potential to greatly enhance their well-being and quality of life since it allows them to monitor their progress and have greater control and responsibility for their daily self-management tasks. The ability to access greater insight, achieve validation, and be supported by authoritative expression in clinical conversations was highly prized. One participant shared how the app can potentially act as an ADHD syndrome tracker, offering her a new perspective on managing her condition. Equally, the ability to feel more in charge of their own wellbeing, and to be supported by ready access to lifestyle and wellbeing management guidance was perceived to be of value for at least seven out of nine participants.

- *Privacy and Data Security*

Even beyond general fears about data breaches, the ongoing controversies and potential prejudice associated with their ADHD condition inspired heightened sensitivity in regard to privacy and data security considerations. This was a repeated concern expressed by seven out of nine interviewees, so a robust and transparent privacy and data security policy impacts what digital tools consumer/service innovation participants choose to use and how they are applied.

- *Tailoring*

Consumer technical know-how and lifestyle preferences varied widely amongst even this small sample group. Whereas some relied heavily on automated reminders to self-organize themselves, others found them annoying, potentially even depressing. Ultimately, the ability to customise the app’s settings emerged as an important consideration. Similarly, ways to track more complex treatment regimens and nuanced life experiences were of interest, particularly given the potentially reductive nature of symptom trackers. Some participants also talked about wanting variable theme options, the ability to adjust the font size, and different layout choices, as well as interactive elements such as interactive charts, feedback options, and custom alerts.

- *Access*

Eight out of nine participants valued the additional accessible support of a mobile app for ADHD management. For care consumers without a car, single mothers, or clients with social anxiety, the app was perceived as a vital tool for those on ADHD medication, facilitating close monitoring to help optimise treatment. The potential benefits of learning how to manage treatments, including both stimulant and non-stimulant medications were regularly discussed, considering the need to adjust dosage over time due to side effects, built-up tolerance, hormonal changes, and shifting symptoms.

App interface/design was an equally important access consideration. An intuitive and user-friendly interface can help to ensure accessibility for care consumers regardless of their tech proficiency. Visual appeal was an important consideration, with at least a third of consumers highlighting their preference for visual rather than textual information. However, participants in this study highlighted several concerns, including confusion when faced with more complex graphics. For example, most participants were confused by the portrayal of wellbeing progression via a prototype spider-web graphic. At least six participants pointed out the need for more streamlining of the interface.

### 3.2. Health Professionals’ Discussion Themes

#### 3.2.1. Contextual Barriers to Better ADHD Care Identified by Health Professionals

Example quotes listed in Table 4. below indicate that health practitioners highlighted the value of this prototype ADHD service innovation, as well as the complex considerations that are essential for integrating these tools effectively in the ADHD care landscape.

- *Complexity*

As in previous studies [26,27,30,31], health practitioners acknowledged the complexity of the ADHD treatment context. In addition to concerns regarding the administrative burden of ADHD diagnosis and treatment cited above, one GP confessed that they did not completely understand the condition. For that reason, the ADHD service innovation was generally welcomed as a potential beacon for change.

- *Sustainability*

Another challenge particularly relevant to the development of the ADHD app is ensuring its long-term relevance and utility. Health practitioners worry that ADHD care consumers will find it hard to stick to their treatment over time. A further consideration is that, beyond diagnosis, treatment pathways can be complex. One clinician shared their concern that consumer/service innovation participants might become dependent or anxious about the expectation of daily check-ins. Reflecting current research thinking, several practitioners confirmed their belief that a comprehensive approach involving medication, condition assessment, lifestyle choices, and psychosocial assessments is generally required to deliver true patient-centred care.

#### 3.2.2. Potential Enablers of the ADHD Service Innovation Identified by Health Professionals

For the optimal utilisation of digital health tools like the app, attributes such as user-friendliness, accessibility, and patient privacy are emphasised by the participants.

In the following section, participant responses to the prototype ADHD service innovation, including identified enablers such as clinical integration, addressing the complexity of the ADHD condition, and ensuring the sustainability of digital tools in treatment regimens is discussed. Example quotes are listed in Table 5 below.

To help manage these challenges, clinicians shared some strategies that they might use themselves to maintain care consumer engagement over time, such as techniques to workshop the care consumer’s lifegoals, reflect upon goal achievement, and embed reminders of those goals in their daily life to ensure that the work required to maintain their own self-care remains meaningful.

- *Transparent Privacy and Security Frameworks*

Healthcare practitioners were sensitive to the importance of transparent privacy and security frameworks for healthcare data, emphasising the foundational aspect of these considerations regarding the potential for engagement in the first place. Requests were made for upfront reassurances in this regard.

- *Streamlining*

Participants in the study underscored how service innovations such as this can help to deliver enhanced, individualised care for people with ADHD. One-stop shop service aids which offered comprehensive support, alongside one-click operations that effortlessly tracked, automatically analysed, and highlighted changes in the consumer’s health and wellbeing were particularly prized. Clinicians expressed an expectation that fit-for-purpose tools can encourage a patient-centred approach.

While the potential for improved communication and patient monitoring is acknowledged, concerns about the added pressures on practitioners and the feasibility of incorporating these tools into existing systems persist. At least one practitioner suggested that it is the patient’s responsibility to take care of their health outside of medical settings.

- *Connected Care*

Linked to the importance of streamlining, the value of shared clinical care was readily acknowledged. Thus, the seamless integration of digital technologies and tools into the standard procedures of general practitioners and other healthcare professionals is viewed as a significant stride toward enhanced individualised ADHD care. Though opinions vary on the optimal implementation due to concerns about additional clinical burden, nevertheless, the value of support for streamlined information exchange between ADHD care consumers/service innovation participants, GPs, and the psychiatric team was readily acknowledged.

- *Wishlist/Need for Tailoring*

Due to the complexity of the ADHD experience, all practitioner participants expressed keen interest in understanding the intricate details of ADHD care consumer’s profiles across multiple domains, including any co-morbid conditions, to tailor care which addresses the individual’s unique needs and challenges.
*Recommended Social and Lifestyle Metrics**To comprehensively manage and support individuals with ADHD, several clinicians recommended that the app be expanded to embrace a holistic approach that delves deep into various facets of their lives.*-*Interpersonal Relationships: Quality of Personal Relationships*-*Emotional Health: Emotional Well-Being*-*Living Conditions: Housing Stability*-*Personal Fulfillment: Self-Satisfaction Levels*-*Nutrition and Consumption: Dietary Habits, Sugar and Coffee Intake*-*Physical Activity: Exercise and sports involvement*-*Psychological Experiences: Recent Traumas*-*Professional Life: Employment Status, Job Security, Workplace Stress, and Satisfaction*

This motivation to engage with a broader view of the care consumer’s wellbeing is tempered by a keen awareness that care consumers seeking treatment support for ADHD are likely to be impatient with long questionnaires, particularly repetitive questions.

The prototype app currently mandates that ADHD care consumers address core questions across ADHD, including impulsivity/hyperactivity and focus, plus broader mental health areas. Care consumers/participants can explore anxiety, stress, and mood further, ensuring a thorough evaluation with minimal flexibility in question responses. In contrast, one practitioner pointed to the value of open customization. They cited the case of a care consumer whose recovery was charted by their ability to independently shop in shopping centres. Furthermore, since clinicians sometimes prefer to address specific issues intensively to bring about immediate relief and noticeable improvement, it was felt that the ability to highlight one or two core symptoms that are most critical to the patient’s current condition would aid focus.

Additional mHealth tips from ADHD practitioners (such as flexible monitoring demands, reminders grounded in daily life like desktop wallpapers, and links to meaningful goals), indicate the value of scalable design and the continuous gathering and analysis of user feedback as essential strategies to achieve this objective, ensuring that the tools and approaches employed are adaptive and responsive to the dynamic needs of ADHD care consumers.

## 4. Discussion

This preparatory investigation reinforces earlier findings [16,63] that the ADHD health care context is in urgent need of innovation.

Contextual barriers to better ADHD care, as identified by consumers, include delayed diagnosis and the lack of readily available, authoritative information about the condition. In the face of potential social stigma, clinical validation and understanding are important considerations. Related to this, curiosity arose regarding the origin of questions and the entities responsible for creating them. Consumers also held concerns about digital security and the practical implications of consistent data gathering. Some participants struggled with technology, especially assessments. These insights highlight the multifaceted challenges faced by individuals seeking better ADHD care.

Overall, a mobile self-monitoring/ADHD guidance tool was seen as a potential enhancer of well-being and quality of life, offering insight, validation, and the opportunity for control. Potential enablers of such a health service innovation included transparent and secure privacy protection and data security. Participants stressed the significance of inclusive language, diagnostic validation, and clear clinical pathways due to past experiences of ignorance and prejudice regarding their ADHD. Customisation options were valued to accommodate diverse preferences and needs. Access to support via mobile apps was also vital, particularly for those with transportation issues or social anxiety. Interface design was crucial for accessibility, with visual appeal and simplicity being key considerations.

Health professionals acknowledged the complexity of ADHD treatment, with concerns about administrative burdens and a limited understanding of the condition amongst GPs. The ADHD mHealth service innovation was welcomed for its potential to address these challenges. Ensuring the long-term relevance and utility of the ADHD app and related information processing and delivery pathways is crucial, considering potential adherence issues and the complexity of treatment pathways.

Health professionals stressed the importance of accessibility, ease of use, customisation and patient privacy for the optimal utilisation of digital health service innovation. The importance of enablers such as clinical integration, as well as transparent privacy and data security frameworks, were highlighted. Clinicians suggested potential strategies to maintain consumer engagement, emphasising valuable add-ons like coaching and a holistic treatment approach. Although concerns about added pressures persist, the integration of digital tools into standard procedures was seen as vital for enhanced ADHD care. Tailoring care to individual needs and challenges was considered essential due to the complexity of the ADHD condition. Streamlined services were valued as a pathway for delivery of more individualised care.

ADHD is a profoundly impactful condition, which is slowly gaining clinical recognition, yet many people are not receiving the diagnosis and treatment that they need. Current ADHD diagnosis and treatment guidelines require expert screening for co-morbid conditions [64,65], but as several of the participants of this study attest, the inter-relationships of these co-morbidities are not always well understood by non-specialists. Substance use disorder, anxiety, depression, alienation, and personality disorders are common co-morbidity experiences [62] amongst people who (as this study reflects) can go years without a parallel, and, in some cases, perhaps even without an underlying ADHD diagnosis, or effective treatment. Given the profound impact of such a gap, there is a need to both streamline and strengthen ADHD care pathways for consumers and healthcare practitioners.

### 4.1. CFIR Construct: Outer Setting

The outer setting context for this multi-site, multi-stakeholder study is characterised by care access bottlenecks and contested treatments, inspiring a 2023 Australian senate enquiry to recommend that efforts be made to find coordinated solutions that can work to promote greater awareness of neurodiversity, broaden access to affordable care, and at the same time, improve the quality of care being accessed [16]. MHealth innovations show great promise in this regard, since their use of accessible, flexible mobile technologies that ADHD care consumers/service innovation participants can tailor to their needs can help to reconfigure ADHD care resources.

#### Implications for the Desired Service Innovation Based upon the Outer Setting

Enabling consumer access to a new co-created mHealth innovations for strengthened self-management, and fine-grained clinical decision support can potentially increase consumer access to responsive clinical guidance [51,66,67,68,69,70]. Equally, there is clinical value in mHealth service innovations that provide a networked pathway for ADHD treatment oversight and collaboration between consumers, psychiatrists, and GPs.

### 4.2. CFIR Construct: Participant Characteristics

There are many implications for the desired service innovation based upon ADHD Care Consumer Characteristics. The value of empowerment and the need for a sense of security are repeated themes in consumer interviews, highlighting the importance of clinical validation in the contested context of ADHD diagnosis and care [13,15,17,18]. Inclusive communication, as much as security and privacy measures, are central considerations.

Empowering individuals to take control of their ADHD condition is an essential strategy to help to ensure the sustainability of self-management efforts. The experience of empowerment, coupled with the ability to access meaningful support and responsive feedback can potentially help to sustain consumer engagement over time [51,66,67,68,69,70]—provided that self-monitoring activities continue to be perceived as meaningful and/or valuable, and that there are enough technical and emotional supports on hand to ensure that ADHD care consumers are not burdened even further by these options [71,72]. This caveat reflects earlier findings that mHealth technologies can bridge service gaps for disadvantaged consumers, but require a networked mix of technical and non-technical resource support for success [70]. Such additional supports are particularly valuable for those ADHD care consumers who struggle to sustain their engagement with a care service over time, and this is discussed in more detail below.

#### Implications for the Desired Service Innovation Based upon ADHD Practitioner Characteristics

Whilst ADHD practitioners generally welcomed care consumer participant monitored data, many expressed a need for more education and support in this context themselves. Concerns about the added pressures on practitioners and the feasibility of incorporating these tools into existing systems persist. Importantly, practitioners point out that the comprehensive care of ADHD care consumers necessitates an individualised approach that not only addresses core symptoms, but also considers comorbid conditions, mental health, and social aspects of care consumers’ lives. Priority areas for innovation include ready access to authoritative oversight, and assistance to help process the administrative burden of ADHD pharmacological treatment, which normally involves a two-to-three-month titration period when different pharmaceuticals and dosages may be trailed to find the best solution for each individual. A streamlined, one-stop-shop solution that could help to smooth the management of ADHD diagnosis and care by seamlessly connecting information flows between all relevant ADHD healthcare practitioners and consumers was of great interest. Practitioners shared concerns about privacy and security, emphasising the need for transparency in this regard, and quickly flagged potential sustainability challenges regarding continued ADHD care consumer engagement over time.

### 4.3. Service Innovation Characteristics

The service innovation held great interest for nearly all study participants and the app was recognised for its intuitive design. Nevertheless, there was also demand for broader metrics and extended features. A tension emerged between the need for more streamlined, pared back content, and a parallel desire for personalised and visually engaging elements which cater for diverse user preferences and needs. Thus, while the prototype ADHD service innovation is acknowledged for its potential to enhance adult ADHD consumer care through networked communication and monitoring functionalities, hurdles related to its practical integration, adaptability, and long-term sustainability are evident. This reflects findings from previous studies [73,74] indicating that clean displays that prioritize the visual communication of information, supplemented with brief textual explanations, provide an important design strategy for mental health apps to help balance these tensions.

#### Implications for the Desired Service Innovation Based upon the Prototype Design

Self-monitoring is likely to be a priority value for participants during the early titration phase of treatment when new prescriptions and medication dosages are being trialled. Beyond the titration phase, ADHD care consumer participants of this study indicated a strong interest in a broad range of healthcare options, including those that invited them to take a more proactive involvement in their own pharmacological treatment, supplemented by access to alternative treatment pathways. Optimum supports include interactional resources such as shared knowledge and streamlined information flows amongst consumers and their networked care providers [70].

Equally, self-monitoring tools which strike a balance between complexity and simplicity, while also offering meaningful insights and some form of psychosocial support, can help to keep consumers motivated and committed to utilising the service for an extended period of time [75]. This proviso reflects earlier findings about the potential burden of self-monitoring consumer technologies [76], and the need for simple, easy-to-use mental health apps [28,77]. Since the technical proficiency of participants varies, alongside patience levels, it is recommended that self-monitoring tasks and additional security measures follow established practice and streamline consumer engagement as much as possible beyond the initial set-up. As noted by an ADHD coach participant in this study, links to everyday interactions and interpersonal supports can help to embed self-management practices within daily life. For sustainability, previous studies also suggest that it can be helpful to supplement services with motivational content, goal setting tools, and achievement rewards, perhaps linked to simple, strategic game-play [74].

As the ADHD prototype app develops greater functionality over time, a central hub may be essential to help streamline participant navigation between discrete functions such as ADHD treatment outcome monitoring versus access to more varied support pathways [75], such as coaching and peer support services. As each consumer participant of this study attested, sharing issues, advice, and solutions with a network of understanding peers and professionals can offer crucial support for individuals managing ADHD. Finding ways to ensure that it is feasible, yet effective, to offer this higher level of support may be a priority consideration for future industry-focused digital health research projects. Additional supports may also potentially include chatbots, which recent studies indicate can offer valuable support alternatives for ADHD sufferers [73,74,78].To further build on the service innovation prototype’s existing strengths, design recommendations are summarised in Table 6 below.

## 5. Limitations

This study has some limitations. In qualitative research, the small sample size used in this study is often appropriate as it provides in-depth nuanced insights into the complex research topic. However, thematical saturation may not have been reached, and a larger sample size may reveal further issues not identified here that are, nevertheless, relevant to the ADHD experience of variable age groups, socio-cultural backgrounds, and clinical groups. The findings may lack generalisability and are typically not meant to be extrapolated to the broader population due to the specific contextual nature of qualitative data.

The generalisability of this study is also limited by lack of functional access to the prototype mobile app during the study period. The availability of a working prototype and the limited interview time with participants, particularly healthcare practitioners, meant that interaction with the ADHD service innovation focused upon a visual review of the prototype screens. Participants did not have the opportunity to explore its features in their own time. The findings are, therefore, indicative and exploratory. However, future research in collaboration with the ADHD service innovation deployment and implementation will be required for more in-depth usability insights.

These exploratory findings reflect the South Australian context where, as previously noted, the pathways for adult ADHD diagnosis and treatment are strictly controlled and a shortage of ADHD treating psychiatrists creates ADHD care bottlenecks. Nevertheless, challenges related to the contested contexts of ADHD diagnosis and care are encountered worldwide [17,63] and, therefore, these findings, particularly with reference to the perceived need for care consumer empowerment and validation, secure and private information flows, streamlined, and collaborative ADHD care can help to guide future research on developing ADHD Service Innovations more broadly.

## 6. Conclusions

ADHD in adults is a prevalent disorder linked to substantial impairments, including occupational, academic, neuropsychological, and social functioning. However, its underdiagnosis and the barriers to ready treatment remain a global challenge. By leveraging the combined insights of ADHD care consumers and practitioners, this co-design study provides a pioneering direction on how and what to include in the prototype development of an adult ADHD digital service innovation that is streamlined, yet also supportive and enabling.

Digital health service innovations, including mHealth self-monitoring tools, can significantly enhance future ADHD care services by providing more accessible assessment and treatment pathways and actively involving consumers in their mental health care. Grounded in real-world experiences and perceptions of both consumers and health professionals, this study illuminates the potential strengths and areas necessitating the refinement of an ADHD service innovation to optimise its value in the shared holistic management of ADHD care. Understanding service gaps is a crucial step to assessing the potential influence and optimal enhancements of this transformational approach to adult ADHD care. Our findings indicate that the secure collection, sharing, and review of consumer self-monitored data in a streamlined manner, involving a collaborative clinical care team that includes the consumers themselves, is essential for enabling ADHD service innovation.

Consumer study participants expressed an intricate blend of optimism and reservations. While the empowerment derived from real-time data and insights on mental health was seen as invaluable, concerns about privacy and data security, the necessity for personalized user experiences, and the depth of content validity underscored the need for a nuanced approach to app development. These findings highlight that adults with an ADHD diagnosis want access to tools to better understand and manage their own wellbeing. A tool that can provide insights and articulate the clinical context of participant’s experience was particularly prized. ADHD care consumers genuinely wanted to learn more about their condition by accessing clear, yet authoritative, guidance which is inclusive and supportive. Within that context, it appears that support and reassurance from peers is often likely to be as important as expert advice.

Health professionals offered a complementary perspective, highlighting the importance of seamless integration of the prototype service innovation into clinical care, as well as the need for the self-monitoring app to adapt to diverse patient needs, and the likely challenges ahead in sustaining participant engagement when the ADHD care consumer’s treatment regime stabilises and/or their needs change and evolve over time. The balancing act between technological innovation and traditional care models emerged as a pivotal aspect, underpinning the app’s potential success or limitations.

In regard to the future trajectory of the ADHD service innovation, the incorporation of feedback, technological refinement, and continuous engagement with ADHD care consumers and professionals is imperative to help craft an experience that is empathetic, responsive, and empowering.

## Figures and Tables

**Table 1:** Summary of demographic information of Adelaide (S.A.)-based interview participants (n = 15).

Participants	Participants Position/Role	Gender (Male, Female, Nonbinary)
P1	Consumer	M
P2	Consumer	NB
P3	Consumer	M
P4	Consumer	M
P5	Consumer	F
P6	Consumer	F
P7	Consumer	M
P8	Consumer	F
P9	Consumer	F
P10	Psychiatrist	M
P11	Mental Health Nurse	F
P12	Psychiatrist	M
P13	General practitioner	M
P14	General practitioner	M
P15	OT/ADHD coach	F
N = 15		F:6 M: 8 NB:1

**Table 2:** Example Quotes to Illustrate Consumer Perspectives of Thematic Contextual Barriers to Better ADHD Care.

Theme	Example Quotes
Ignorance and Prejudice	*(The lack of diagnosis over many years) made me feel a bit cross. … You know, I would have thought that … this information is readily available now. Maybe it just wasn’t readily available 10 years ago, I don’t know. … it just would have meant understanding myself a lot more. It would have given me a bit more compassion and you know, a little bit more awareness about understanding what it is that makes the makes me experience the world the way I do.* P9 *I’ve tried multiple people. Some GPs dismissed me, saying, “You wouldn’t have ADHD, otherwise you wouldn’t be able to manage to work and all that”.* P1 *So when I first went to my GP, things didn’t go as smoothly as I hoped. I tried explaining everything—how I felt, the symptoms, everything I read about ADHD. But because of my past with substance abuse and alcohol, my GP kinda brushed it off at first. … I felt super frustrated and defeated.* P4 *Wikipedia and YouTube are good for … science topics like physics and electrical engineering, but not for mental health. I find there’s a lot of pop psychology on there, and it’s not always accurate.* P1 *Going online … at first, it felt awesome because so much clicked, like, “That’s me!” But the more I dug, the more overwhelmed I got. There’s so much info, and it’s not all clear or trustworthy.* P4
Trust	“… are any of us safe in this digital world? It feels like our security is hanging by a thread sometimes”. P6 Wow, this sounds really promising! But I’m wondering, what comes after gathering all this data? How are we going to use it effectively? I mean, if I’m consistently checking in and answering questions about how I’m feeling, I worry it might heighten my anxiety or stress. It feels like I’d be concentrating on the negative emotions or symptoms, you know? P8Wow, this sounds really promising! But I’m wondering, what comes after gathering all this data? How are we going to use it effectively? I mean, if I’m consistently checking in and answering questions about how I’m feeling, I worry it might heighten my anxiety or stress. It feels like I’d be concentrating on the negative emotions or symptoms, you know? P8 *Where are these questions coming from, and who’s behind creating them?* P7
Impatience	*“I struggle with a lot of it(technology), particularly things like assessments that are supposed to be enjoyable. Yeah, no, kidding. I forget they exist. I get annoyed at the reminders because I do get pathological demand avoidance, where … as soon as it told me I have to do It, I can’t”.* P2 *… Previously, I kept my distance from technology; it was more stressful than helpful. Getting medication reminders four times a day would make me nervous. It felt like a constant reminder of my ADHD and other illness. Seeing all my symptoms written on one page? That is overwhelming for me.* P8 *The app has clean layouts, intuitive icons, and straightforward menu options, which I appreciate. However, having too many questions on a single page can be overwhelming and distracting, especially for someone with ADHD like me.* P4 *… Previously, I kept my distance from technology; it was more stressful than helpful. Getting medication reminders four times a day would make me nervous. It felt like a constant reminder of my ADHD and other illness. Seeing all my symptoms written on one page? That is overwhelming for me.* P8 *… having too many questions on a single page can be overwhelming and distracting, especially for someone with ADHD like me.* P4

**Table 3:** Example Quotes to Illustrate Identified Thematic Enablers of the ADHD Service Innovation.

Theme	Example Quotes
Validation/ empowerment	*Oh, that’s awesome! I can share this app and my results with my mom. It’ll show her that it’s not all just in my head!” … When I go to see my GP, it always feels so rushed, and I completely forget what I wanted to share with him. Being able to print the findings report helps me stay focused during our talks. … it is hard for me to express my selves verbally.* P1 *“Getting insights on my health and comparing it with how I was doing last week can be a game changer. It’s like having a reality check. It’s not just about the data; it’s the empowerment that comes with it, making me think twice about my choices. If last week was a good week because I exercised more, it’s pushed me to keep that momentum. And if the app shows my mood’s been all over the place, maybe it’s time to ring up my doctor. It’s like having a personal health assistant in my pocket”.* P2
Privacy and Data Integrity	*“Where is my everyday mood data going? I’m not convinced anyone would be interested in my anxiety, but it’s good to know. … I don’t want even my partner to find out about my anxiety and other issues, especially if he were to randomly use my phone”.* P5 *Talking about my mental health is very tough for me I’ve never been comfortable talking about my depression; I’ve always been that way. But my doctor believes I am depressed.* P7
Tailoring	*I am taking stimulant medication, as well as the sex hormone, and I‘m not sure how this app could capture the combined effects of these medications on my mental health.* P2 *“The questions should resonate with my experiences and challenges. If the tool seems generic or not applicable to my specific situation, I might lose interest”.* P7 *…if it is actually, you know, a true representation of how I’m actually doing, I find my experience with that stuff is … it’s not really very accurate, right? … The nuances of how I’m doing are so complex, and so interconnected to things that have nothing..(and) everything to do with my mental health.* P9 *I get the simplicity, but it’s kind of dull for my taste. Wouldn’t it be cool if we could jazz it up with some colours and icons? Nothing too flashy, of course. And hey, letting us pick our own background colours—like other Apps … Features like voice commands, and easy readability can be helpful. …* P6 *I am taking stimulant medication, as well as the sex hormone, and I‘m not sure how this app could capture the combined effects of these medications on my mental health.* P2
*Access*	*“I don’t like to go out unless I must, and with my family living 200 km away, I’m on my own a lot. This app can be a lifesaver, allowing me to assess myself. It’d be even better if I could log and track my feelings, sleep, and other symptoms to see if my medication is working. And ideally, share it with my GP. … I wish I could communicate with my GP in this app”.* P3 *I‘ve recently been diagnosed with ADHD and started medication. How can I tell if it’s working? I really need support in understanding what to expect from this treatment.* P8

**Table 4:** Example Quotes to Illustrate Health Professional Perspectives of Thematic Contextual Barriers to Better ADHD Care.

Theme	Example Quote
Complexity	*“To be honest, I prefer not to see ADHD patients. There are a few reasons for this. First off, there’s just so much paperwork and documentation. Every time I see an ADHD patient, I need to send their details to DASSA (Drug and Alcohol Services, SA). And then there’s the matter of medications. They’re not straightforward to adjust in terms of dosing, and there’s always this lingering worry about the potential for medication abuse. I‘ve heard of instances where patients take more than they should or their meds end up being used by someone else, maybe a family member or a friend”.* P14 *I never really received proper training for treating ADHD, and I don’t see many patients with the condition to begin with. And, honestly, the legalities also make it tricky. As a GP, I‘m not allowed to diagnose ADHD or start patients on medication. That’s something that needs a psychiatrist’s input. After they give a diagnosis and recommend the right dosage, only then can we move forward with treatment. So, all these factors combined make it a challenge for me.* P14
Sustainability	*“So in terms of stickability the self-reporting needs to have enough sort of reinforcement. Qualities that the patient sees value … if it feels like a chore to use, they won’t stick with it. We want them to feel empowered, not overwhelmed”.* P10 *I would imagine … that after that initial motivation and drive wears off, they might forget that that app maybe exists. So forgetting to check in, you know, maybe their priorities shifts. And that apps no longer meeting their needs. So if I was designing … I‘d probably be looking at regular reminders, which had a consistent time of the day for them to check in. I guess I‘ve been trying to make that app simple as well. So if you’re thinking that potentially you might only captivate their attention for a short span of time, getting the most out of their attention.* P15 *I suppose that one risk I see with these kinds of apps is that … if I have to check on myself all the time … I’m also getting reminded that, well, there are these areas have got problems with, but what often happens when people get really better … they just want to, you know, go on and live life.* P12

**Table 5:** Example Quotes to Illustrate Thematic Enablers of the Prototype ADHD Service Innovation.

Theme	Example Quote
Transparent privacy and security frameworks	*I prefer a very organised approach when seeing patients.* P13 *“Above all, the data has to be secure. Knowing their personal health info is locked down tight would give us peace of mind”.* P14
Streamlining	*I think to make GP’s lock into another system is a pipe dream. … Patients need to own the data, and then if you have a consult with a psychiatrist they can show the data and say, look, that’s how I’ve been tracking.* P10 *“(In an ideal situation) A tool that is secure, user-friendly, and offers features like one-click sharing of data, automatic trend identification, and integration with other health data could make self-monitored records more useful for both patients and practitioners”.* P14
Connected care	*“I believe a collaborative system that involves the general practitioner, patient, and psychiatrist and effectively tracks treatment outcomes would be immensely helpful”.* P14 *… the platform that we use…helps with us scheduling clients having all the client details on there, we can use in case notes but most importantly, it helps us link in with telehealth, send emails to clients and text messages. … its pretty easy for the clients to use as well. When they’re joining telehealth appointments. All they really have to do is click the link put their name in and they join. … I don’t really have any complaints around that database. The other one (A database used elsewhere which the participant describes as clunky), I do have a lot of complaints around … it doesn’t support shared care with other practitioners.* P15
Wishlist/ Need for tailoring	*I think it’s very difficult to create a list that’s relevant to everybody. Because people have very individual areas of their lives that they identify as having problems with. … So, for example, I’ve got one patient who...couldn’t go shopping independently in shopping centers. … But that might not be a thing for you know, the ten other people. … it would be good if it was customizable.* P12 *You know anything that’s a visual scale. So for example, if there was, you know, a total symptom scale that people fill out, but then the total score would be plotted over time. … It would be helpful because then you can see that, you know in one glance...(and) if there’s a report for the first assessments of baseline before treatment, for example, … and if they repeat those scores, it would be good to have that represented as a visual plot. But only for the symptoms that really matter.* P12 *I‘d love a simple way for patients to share their logs or data with me. Perhaps a one-click option that sends records straight to a secured platform? No more waiting for appointments to get updates. Charts and graphs! I‘d want the tool to identify and highlight trends automatically. Like, “Hey, notice more focus issues on Tuesdays?” or “Looks like mood depressions every evening”. That kind of insight can be game-changing … Think of it as a digital companion, not just a journal. Something interactive, insightful, and integrated. That’d be the dream toolkit. P14* *“Their social life: Are they engaged in social activities, and how often?* *Substance use: Are they using any substances?* *Education: What is their educational background and status? How they cope with achieving their educational goals. * *Relationships: What is the nature of their personal relationships? Are they currently in a relationship? Or their emotional life in general. * *Living situation: Are they living with anyone? Is their housing stable?* *I also like to know their level of self-satisfaction. Are they making personal progress when compared to their past selves?”* P14

**Table 6:** Summary of The Interview Findings and Recommendation for Improvement.

Key Features of The ADHD Service Innovation Advantages of the Prototype ADHD Service Innovation	
1. Alignment with Established Guidelines The ADHD service innovation aligns with established care principles that highlight the key role of self-management in chronic care [79], and incorporates validated standards (linked to clinical oversight) and content. 2. Comprehensive Assessment The service innovation evaluates a care consumer’s mental and emotional well-being in real-time employing Ecological Momentary Assessments, offering a comprehensive yet efficient means of tracking mental health. 3. Data Insight The ADHD service innovation can empower care consumers by offering insights into their mental health and enables them to make informed decisions to manage their mental health effectively. 4. Extra support The ADHD service innovation which fosters self-monitoring of treatment outcomes, complemented by the accompanying self-monitoring report, is designed to facilitate enhanced self-management by integrating educational content and resources directly within the app. This addition can enrich the care consumer’s knowledge and understanding of ADHD and mental health whilst equipping them with practical self-management strategies.	
Area For Enhancement 1: Privacy and Confidentiality Some participants with co-occurring mental health conditions expressed discomfort in discussing and recording sensitive information related to their mental health on the app. Likewise, health practitioners flagged privacy and confidentiality as important considerations.	
	Recommendations Implement a systematic mix of technologies and best practices such as technical de-identification of data, restrictive data access, and security measures in the underlying technical platforms. Strengthen privacy features and clearly communicate them to care consumers. Clearly outline the purposes, uses, and privacy protections related to collected data. Implement a transparent communication strategy to educate ADHD care consumers on data handling and protections to build trust. Implement additional security measures, if necessary, like optional multifactor, or biometric authentication and encryption to ensure data security and user confidentiality.
Area For Enhancement 2: Tailoring: A one-size-fits-all approach might not cater to consumers with diverse and complex needs. Tailored experiences based on individual user needs, preferences, and mental health conditions enable streamlining and greater focus upon priority metrics, as required. Allowing ADHD care consumers to personalize question types, frequency, and focus areas will enhance the app’s usability. Enabling care consumers to tailor the app’s appearance to their preferences, can enhance visual appeal and user engagement.	
	Recommendations Allow care consumers to personalize question types, frequency, and focus areas. Enhance the app’s adaptability to offer tailored experiences based on individual consumer needs, preferences, and mental health conditions.
Area For Enhancement 3: Sustainability App Interface: Tensions between the need for simplicity versus the need for sustained engagement. Not all care consumers are equally tech-savvy, and so ease of engagement, as much as the attraction of additional features are equal considerations to engage this (potentially impatient) audience and maximise the use of the newly developed functionalities. To help maintain the engagement of this population group it is preferable if questions are kept short, screen content is simplified, visuals and language are pro-actively inclusive, sensory engagement is heightened, personalisation features are enabled, and participants are provided ample guidance and support throughout the onboarding process.	
	Recommendations The service innovation held great appeal and interest for most participants. Enhancing it with the following features could increase its appeal even further, especially considering the ADHD community’s need for visual stimulation and engagement to maintain interest: Streamline Content: Keep questions short. Simplify screen content. Emphasize inclusion: Use inclusive language, relatable imagery, and real stories from other people with lived experience of ADHD. Sensory Engagement: Integrate elements of music or art, voice recording, calendars, note-taking to enrich the user experience, ensuring ADHD care consumers feel positive and engaged while using the app. User Onboarding: Implement intuitive ‘How-To’ screens following the initial download and subsequent updates to guide care consumer’s effortlessly through the app’s features and functionalities. Explain the metrics rationale, perhaps with a contextual ‘i’ (information) graphic next to each question, and/or a ‘Why am I being asked this?’ link on relevant pages. Personalization Features: Offer customisable background colours and fonts, allowing care consumers to tailor the app’s appearance to their preferences, enhancing visual appeal and user engagement. This facility also supports streamlining of the monitoring functionality to enable greater focus upon priority metrics.
Area For Enhancement 4: Robust Usability Feedback Mechanisms Frameworks for app user experience feedback and technical support within the app (or separately) are recommended to enable participants to share insights, experiences, and suggestions for improvements. Over time, this feedback can continue to guide iterative enhancements to the app’s content and features for sustainable improvement. Features that also allow participants the feeling and experience of connecting with others, by sharing experiences and gaining interpersonal support in managing ADHD offer significant value for this population group.	
	Recommendations Allow care consumers to personalise question types, frequency, and focus areas. Enhance the app’s adaptability to offer tailored experiences based on individual consumer needs, preferences, and mental health conditions.
Area For Enhancement 5: Potential Future Developments To sustain consumer engagement in the long term, the continued utility and value of such a service will need to be readily apparent even after treatment has stabilised. Recommendations for added value enhancements include the provision of insight and coaching. The ADHD service innovation is in the early stages of development. Nevertheless, a range of advanced features were identified to be of interest.	
	Recommendations Consider the inclusion of optional location-based triggers for the benefit of consumer participants, so that they can set reminders when they leave their home or the workplace. To help ensure sustainability and extend the reach of the service innovation beyond titration (both before and after diagnosis) consider the provision of either AI enhanced chat, or telehealth links to ADHD lifestyle coaching including tailored goal setting, positive reinforcement (numerous quick wins), and lifestyle management tips in response to a broad range of user monitored data. Alternatively, consider developing a virtual ADHD coach. This avatar feature can provide emotional support, basic medical guidance, and connections to other services. If open text commentary is enabled for nuanced monitoring, offer consumer participants AI enhanced sentiment-analysis to reward engagement and the option to either keep that content entirely private, or only share an overview of the sentiment analysis with practitioners

## Data Availability

The data presented in this study are not shared publicly due to privacy and ethical restrictions.
